# Dispersion and Dosimetric Challenges of Hydrophobic Carbon-Based Nanoparticles in In Vitro Cellular Studies

**DOI:** 10.3390/nano14070589

**Published:** 2024-03-27

**Authors:** Denisa Lizonova, Una Trivanovic, Philip Demokritou, Georgios A. Kelesidis

**Affiliations:** 1 Nanoscience and Advanced Materials Center (NAMC), Environmental and Occupational Health Science Institute, School of Public Health, Rutgers University, 170 Frelinghuysen Road, Piscataway, NJ 08854, USA; 2 Particle Technology Laboratory, Institute of Energy and Process Engineering, Department of Mechanical and Process Engineering, ETH Zürich, Sonneggstrasse 3, CH-8092 Zürich, Switzerland

**Keywords:** hydrophobic nanoparticles, black carbon, dispersion, protein stabilization, cell culture, in vitro testing, effective density, dosimetry

## Abstract

Methodologies across the dispersion preparation, characterization, and cellular dosimetry of hydrophilic nanoparticles (NPs) have been developed and used extensively in the field of nanotoxicology. However, hydrophobic NPs pose a challenge for dispersion in aqueous culture media using conventional methods that include sonication followed by mixing in the culture medium of interest and cellular dosimetry. In this study, a robust methodology for the preparation of stable dispersions of hydrophobic NPs for cellular studies is developed by introducing continuous energy over time via stirring in the culture medium followed by dispersion characterization and cellular dosimetry. The stirring energy and the presence of proteins in the culture medium result in the formation of a protein corona around the NPs, stabilizing their dispersion, which can be used for in vitro cellular studies. The identification of the optimal stirring time is crucial for achieving dispersion and stability. This is assessed through a comprehensive stability testing protocol employing dynamic light scattering to evaluate the particle size distribution stability and polydispersity. Additionally, the effective density of the NPs is obtained for the stable NP dispersions using the volumetric centrifugation method, while cellular dosimetry calculations are done using available cellular computational modeling, mirroring approaches used for hydrophilic NPs. The robustness of the proposed dispersion approach is showcased using a highly hydrophobic NP model (black carbon NPs) and two culture media, RPMI medium and SABM, that are widely used in cellular studies. The proposed approach for the dispersion of hydrophobic NPs results in stable dispersions in both culture media used here. The NP effective density of 1.03–1.07 g/cm^3^ measured here for black carbon NPs is close to the culture media density, resulting in slow deposition on the cells over time. So, the present methodology for dispersion and dosimetry of hydrophobic NPs is essential for the design of dose–response studies and overcoming the challenges imposed by slow particle deposition.

## 1. Introduction

Engineered nanoparticles (NPs) are finding their way into many products and applications in various fields, including cosmetics, catalysts [1], sensors [2,3], medicine [4,5], as well as in agriculture and food areas [6,7], leading to inevitable human exposure [8,9]. Moreover, various environmental NP pollutants undergo undesired release to the environment during the manufacturing of nano-enabled products [10,11,12,13] or as a by-product of fossil fuel combustion in diesel [14,15], marine [16,17], and aircraft engines [18,19], or wildfires [20,21,22]. 

There is also emerging evidence that nanoscale particles can bypass biological barriers, become systemic [23,24], interfere with cell function and can be bioactive and toxic [25,26]. Thus, their effects on biological systems should be quantified [27]. The standard toxicological evaluation of both environmental [28] and engineered [29] NPs requires in vitro studies using cell cultures, wherein the NPs are dispersed in biorelevant solutions containing cells to assess the bioactivity and mechanistic pathways of interest [27,30].

For hydrophilic NPs, such as metal oxides or silica, in the nanotoxicology field, there have been methods developed and standardized across the dispersion preparation–characterization–dosimetry continuum [31,32,33]. The dispersion of hydrophilic NPs takes place first in water at a predetermined critical sonication energy as described by the authors in their previous publications [31,34]. Then, the dispersion is mixed by vortexing in the culture medium of interest, which results in fairly well-dispersed and stable NP dispersions. It is worth noting that direct sonication of NPs in the culture medium is prohibited in order to avoid generation of reactive oxygen species from sonolysis and protein denaturation [35,36,37]. On the other hand, hydrophobic NPs cannot be easily wetted by pure water [38] and, thus, an alternative methodology is needed to prepare stable NP dispersions in culture media for cellular studies. 

In order to properly disperse hydrophobic NPs in water, they have to be chemically modified [39,40] or non-covalently stabilized by various chemical surfactants [40,41] that alter the particle surface chemistry and may affect the interaction between nanoparticles and the biological system, compromising in that way the toxicological endpoint evaluation [42]. Previously, a direct dispersion in cell culture media in combination with sonication was done [43], which may, however, affect proteins in the media due to sonication-induced aggregation [44] and also generate reactive oxygen species via sonolysis and protein denaturation [35].

In this study, we introduce a methodology for dispersing hydrophobic NPs directly in cell culture media without using chemical surfactants and sonication. The methodology builds upon the work described in detail by DeLoid et al. [34]. So, a sonication step is substituted by continuous stirring in the presence of proteins, such as fetal bovine serum (FBS) or bovine serum albumin (BSA), that are present in the cell culture media. The proteins adsorb on the surface of hydrophobic NPs mostly by hydrophobic interactions [45,46,47] and form a protein corona [48,49,50,51]. This enables the formation of stable dispersions in water that can be used in cellular studies, such as cytotoxicity testing or investigation of various toxicological endpoints, which is essential for the toxicological evaluation of hydrophobic nanoparticles. Adsorption of proteins is governed by their chemical identity, as well as by the nanoparticle surface properties [48,50,51]. It is well known that proteins readily adsorb on hydrophobic nanoparticles [52]. Therefore, the methodology presented here for the dispersion of nanoparticles in cell culture media will be effective for the vast majority of hydrophobic nanomaterials. Stabilization of dispersions of hydrophobic NPs using proteins has been previously investigated for microplastics [53]. The robustness of the method is showcased here using model hydrophobic black carbon (BC) NPs and two types of cell culture media, which differ in protein content: RPMI medium supplemented with 10% FBS, and SABM supplemented with 1% BSA. 

## 2. Methods

### 2.1. Summary of the Dispersion Preparation and Characterization Approach for Hydrophobic NPs

**Figure 1:**
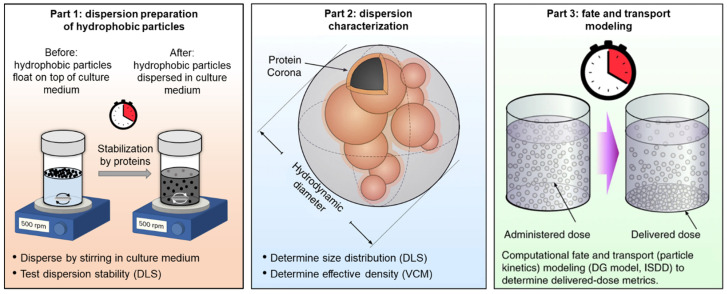
Overview of hydrophobic particle dispersion preparation. An adjusted methodology enabling the preparation of dispersions of hydrophobic particles in biorelevant media suitable for in vitro toxicology studies (**Part 1**). The dispersion prepared that way is further characterized by dynamic light scattering (DLS) to obtain the NP hydrodynamic diameter, and by the volumetric centrifugation method (VCM) to obtain the effective density (**Part 2**), while computational fate and transport modeling is used to determine the dosimetry (**Part 3**). Adapted with permission from DeLoid et al. [34].

Figure 1 illustrates the proposed approach of preparing, characterizing, and modeling dosimetry for hydrophobic NPs. To create a stable dispersion suitable for toxicological evaluations in cell cultures, a protocol involving stirring NPs over time directly in a culture medium enriched with proteins is described here (Part 1).

Proteins adsorbed on the surface of these NPs mostly through hydrophobic interactions, creating a protein corona [51] that enabled the NPs to suspend in an aqueous environment. The resulting NP dispersion was then characterized in detail using dynamic light scattering (DLS) to obtain the particle size distribution and its stability over time, and the volumetric centrifugation method (VCM) to obtain the effective density of the formed agglomerates (Part 2). Lastly, fate and transport modeling was used in order to gather information about dosimetry and NP deposition to cells as a function of exposure time (Part 3). 

The proposed approach was validated experimentally using a hydrophobic NP model, namely the BC NPs described in Section 2.2, and two widely used cell culture media as described below. The effect of stirring energy and time was also investigated in this case study as described in detail below.

### 2.2. Synthesis and Contact Angle Characterization of Hydrophobic Black Carbon NPs

Hydrophobic BC NPs were generated by enclosed spray combustion of jet A fuel at an effective equivalence ratio of 1.77 as described by the authors in their previous publication [15]. The morphology, composition, nanostructure, and primary particle size distributions of the BC NPs emitted by the reactor are in excellent agreement with those measured from real aircraft engines [54,55]. 

The hydrophobicity characterization of the BC NPs was done using a custom-made goniometer following the procedure proposed by Lamour et al. [56]. In brief, BC was placed on a glass fiber filter, a hydrophilic substrate, and then pressed to create a flat and even surface. Then, a drop of deionized water was placed on the pressed BC layer with a syringe. A photo was taken and the water contact angle (WCA) was estimated by analyzing the image using Image [57]. The contact angle measurements obtained using the present goniometer were validated by comparing them to the WCA of a commercial carbon black (Printex 95, Orion Engineered Carbons, Eschborn, Germany) measured with a commercial high-precision optical measuring device (OCA35, DataPhysics, Charlotte, NC, USA). The WCA = 89° ± 5° measured using the custom-made goniometer is in excellent agreement with the WCA of 90° ± 5° obtained using the commercial one (Figure S1), validating the present WCA measurements. 

### 2.3. Model Cell Culture Media Used in the Case Study

Roswell Park Memorial Institute Medium 1640 (RPMI, 1875-093, Gibco, Thermo Fisher Scientific Inc., Waltham, MA, USA) was supplemented with 10% heat-inactivated fetal bovine serum (FBS, 35-011-CV, Cornig, Glendale, AZ, USA), 1% penicillin–streptomycin solution, 1% HEPES buffer, and 1% amphotericin B. Small Airway Basal Medium (SABM, CC3119, Lonza, Morristown, NJ, USA) was supplemented with 1% bovine serum albumin (BSA; CC4162M), 0.1% epinephrine (CC4221M), 0.1% hydrocortisone (CC4031M), 0.1% transferrin (CC4205M), 0.4% mL bovine pituitary extract (CC4002M), 0.1% mL retinoic acid (CC4085M), 0.1% mL epidermal growth factor (CC4230M), 0.1% mL gentamicin sulfate amphotericin B (CC4081M), 0.1% mL insulin (CC401M), and 1% penicillin/streptomycin antibiotics.

### 2.4. Dispersion and Characterization of BC NPs in Cell Culture Media

About 1 mg of BC was placed in a sterile 20 mL scintillation glass vial with a screw top. Subsequently, a Teflon-coated magnetic stirring bar was introduced into the vial, and 10 mL of the selected medium (RPMI + 10% FBS, or SABM + 1% BSA) was added. The sample was vortexed for 10 s using an analog vortex mixer set to a maximum speed. Then, it was placed on a magnetic stirrer adjusted to 500 rpm and stirred at room temperature. To enhance the interaction of hydrophobic NPs with the protein-containing medium and facilitate the stabilization process, the sample was vortexed for 10 s every hour for the first 6 h of the stirring process. Following this period, vortexing was stopped to limit foaming. 

Samples were then stirred at 500 rpm for 6, 12, 24, 48, or 72 h to identify the optimal stirring time. After completing the stirring, the sample was removed from the stirrer, vortexed for 10 s, and 1 mL of the dispersion was collected for particle size distribution analysis using dynamic light scattering (DLS; Zetasizer Nano-ZS, Malvern Panalytical, Westborough, MA, USA). A real refractive index part of 1.66 [58] was used to analyze BC NPs with DLS. The refractive indexes were set to 1.34 and 1.33 for SABM and RPMI medium, respectively, while the dispersant viscosity was set to 1.054 and 0.98 cP, respectively (default dispersant settings). The remaining sample in the original glass vial was stored at room temperature for 24 h to study the stability. Then, it was vortexed for 10 s, and 1 mL was collected and analyzed by DLS. Triplicates were obtained for each DLS measurement to derive the error bars for each particle size distribution. 

### 2.5. Effective Density Measurements and Particle Dosimetry

The effective density, *ρ_eff_*, of dispersed BC NPs in SABM or RPMI medium was obtained following the protocol outlined by DeLoid et al. [59]. In brief, 1 mL samples of 0.1 g/L dispersion were dispersed into TPP PVC tubes (Techno Plastic Products, Trasadingen, Switzerland) and centrifuged at 3000× *g* for 3 h. The agglomerate pellet volumes, *V_pellet_*, were measured using a slide rule-like easy-measure device also obtained from the PCV tube manufacturer. Effective agglomerate densities were calculated from *V_pellet_* values of triplicate samples for each medium according to Equation (1) utilizing a bulk BC density, *ρ_b_*, of 1.8 g/cm^3^, the density of the given culture medium, *ρ_media_*, the mass of BC NPs, *m_BC_*, and a stacking factor, *SF*, of 0.634, i.e., the theoretical value for random close stacking of particle agglomerates [60]:

(1) $$\rho_{eff}=\rho_{media}+\left[\left(\frac{m_{BC}}{V_{pellet}SF}\right)\left(1-\frac{\rho_{media}}{\rho_{b}}\right)\right]$$

The effective density value together with the volume-based particle size distribution of the BC in the cell culture medium were employed to calculate the delivered dose metrics as a function of exposure time using a numerical distorted grid (DG) model [32,61]. The summary of input parameters for the computational modeling is provided in Table S1. It is worth noting that other cellular dosimetry models available can also be used for the dosimetric calculations [33,62]. 

## 3. Results and Discussion

### 3.1. Characterization of BC NPs

The BC NPs used in this study were generated by enclosed spray combustion of jet A fuel [15]. The morphology, composition, nanostructure, and primary particle size distributions of these BC NPs are in excellent agreement with those measured from real aircraft engines [54]. The details on the physicochemical properties of BC NPs used in this study are presented in great detail by the authors in their previous publication ([15]: sample produced at 0% O_2_ concentration). In summary, the BC NP agglomerates consist of primary particles with a mean diameter of 12 nm and a specific surface area of 292.3 m^2^/g. They consist mostly of non-volatile, elemental carbon, as their organic-to-total carbon (OC/TC) mass ratio is just 5.1%. The values of the average interlayer distance, *d* = 3.7 Å, and the average crystallite length, *L_c_* = 1.4 nm, obtained by XRD quantify the graphitic structure of the present BC NPs, which are in agreement with the XRD pattern of unoxidized carbon black [63] and aircraft soot [64]. The Raman spectrum of the BC NPs exhibited a disordered-over-graphitic band ratio, D/G, of 0.93. 

It is worth noting that BC NPs emitted by combustion of fossil fuels, e.g., during road and air transportation [65], are environmentally relevant and have had the interest of toxicologists for quite some time [66,67]. The literature on the biological effects of BC NPs suggests that these can cause cytotoxicity, oxidative stress [68,69], pulmonary tissue damage [70], and may also have an impact on T lymphocytes, indicating potential implications for allergies and cancer development or progression [71]. 

### 3.2. Hydrophobicity of BC NPs

**Figure 2:**
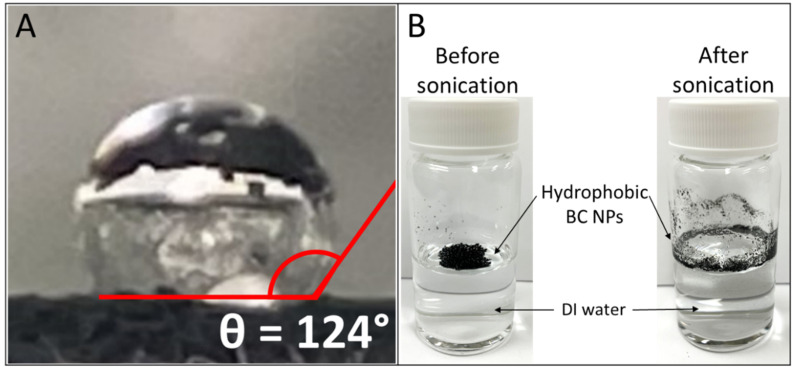
Water contact angle (WCA) measurement of BC NPs using a custom goniometer (**A**) and illustration of their behavior upon contact with water and subsequent sonication as described by the standard dispersion preparation methodology introduced by DeLoid et al. [34] (**B**).

The BC NPs used in this work are highly hydrophobic, as indicated by the large water contact angle (WCA) of 124 ± 2° (Figure 2A). This is within the common WCA range (90–150°) [72] measured for hydrophobic and superhydrophobic NPs, such as diesel BC [73], superhydrophobic fluorosilane silicas [74], and polymer–carbon nanotubes composites [75]. It is worth noting that the standard sonication protocol for dispersing hydrophilic NPs in deionized (DI) water [34] was attempted and was not effective due to the impossibility of wetting hydrophobic particles such as BC NPs (Figure 2B). 

### 3.3. Dispersion Preparation of BC NPs in Cell Culture Media as a Function of Stirring Time (Dispersion Energy)

#### 3.3.1. BC-RPMI Case Study

**Figure 3:**
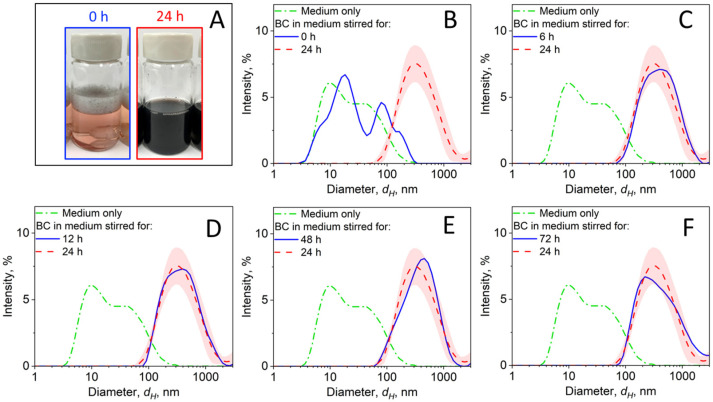
Representative images (**A**) of the dispersions of BC NPs in RPMI medium after stirring for 0 (blue-framed) and 24 h (red-framed), along with intensity-based particle size distributions measured by dynamic light scattering (DLS) for the medium only (dot–broken line) or BC dispersions after 0 (**B**), 6 (**C**), 12 (**D**), 48 (**E**), 72 (**F**), or 24 h (broken line). Variation within three DLS measurements of the sample stirred for 24 h is quantified by the red-shaded area.

Figure 3 shows the evolution of the particle size distribution (PSD) measured for BC NPs in the RPMI medium during the dispersion preparation process as a function of stirring time (dispersion energy). The RPMI medium (Figure 3B–F: medium only, dot–broken line) contains proteins, which were detected by the DLS in the size range of ~5–200 nm. After introducing the BC NPs and vortexing the sample for 10 s, particles start to slowly get into the dispersion. This is reflected in the small shift of the particle size distribution to the larger sizes (Figure 3B, solid line). After 6 h (Figure 3C, solid line) of stirring, the concentration of BC NPs in dispersion increased, and these particles were detected by the DLS in the size range between ~100 and 2000 nm. The PSDs measured after 12 (Figure 3D, solid line), 24 (Figure 3B–F, broken lines and shaded areas), 48 (Figure 3E, solid line), and 72 h (Figure 3F, solid line) of stirring did not vary significantly and are within or close to a standard deviation range, shown here for 24 h with a shaded area. The insets in Figure 3A show representative images of the sample at the beginning of the stirring process (Figure 3A: blue-framed inset) and after 24 h (Figure 3A: red-framed inset), where a color change from a pink to black is attributed to the dispersion of the dark-colored BC NPs in the RPMI medium. Additional images of BC NPs being stirred over time in RPMI medium are provided in Figure S2. 

**Figure 4:**
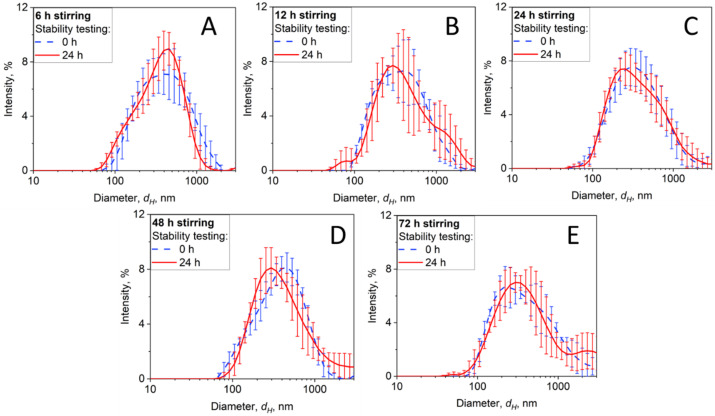
Stability of the BC size distribution measured in RPMI medium right after stirring (broken lines) for 6 (**A**), 12 (**B**), 24 (**C**), 48 (**D**), and 72 h (**E**) or after 24 h of storage at room temperature (solid lines). The error bars quantify the statistical variation over 3 measurements.

The dispersion stability is an important characteristic necessary for its successful application in cell cultures. The preparation method may have a direct effect on the dispersion stability over time and, thus, must be tested. For this purpose, we conducted stability testing of samples stirred for 6–72 h, analyzing changes in the hydrodynamic diameter (Z-Average), the shape of the PSD and its variability throughout the measurement, as well as the polydispersity index (PDI). Figure 4 illustrates the DLS analysis of the dispersion stability of BC NPs in RPMI medium stirred for (A) 6, (B) 12, (C) 24, (D) 48, and (E) 72 h. The Z-Average values together with the PDI values for BC NPs in RPMI are summarized in Table 1. Moreover, the zeta potential and the conductivity of BC NPs dispersion in RPMI medium are provided in Table S2. It should be noted that further studies are needed to understand how the hydrophobicity of such NPs affects the protein corona, which can also result in protein denaturation and further alter the bioactivity [76].

While the differences in the stability between the individual samples shown in Figure 4 are rather minor, the DLS data indicate that the BC NP sample stirred in RPMI medium for 24 h performed the best, as the size distribution did not change significantly after the 24 h stability testing. While lower stirring times were insufficient, resulting in an unstable product characterized by high standard deviations, excessive stirring for too long may also compromise dispersion stability. Specifically, the size distributions at 48 h and 72 h show an increase in the fraction of particles above 1000 nm and higher standard deviations, indicative of possible agglomeration or destabilization. Therefore, it is concluded that for this NP–culture medium system, a 24 h stirring suffices to form a proper and stable dispersion.

#### 3.3.2. BC- SABM Case Study

The DLS measurements of the BC NPs in SABM provided in Figure S3 show similar trends to those described above for the RPMI medium. The proteins in the SABM (Figure S3B–F: medium only, dot–broken line) were detected by the DLS in the size range of ~5–300 nm, showing a multimodal PSD. A slight increase in the bigger size range was observed after introducing the BC NPs into the medium (Figure S3B, solid line). After 6 h of stirring (Figure S3C, solid line), the BC NPs were detected by the DLS in the size range between ~100 and 2000 nm. The PSDs measured after 12 (Figure S3D, solid line), 24 (Figure S3E, solid line), 48 (Figure S3B–F, broken lines and shaded areas), and 72 h (Figure S3F, solid line) exhibit higher variability when compared to the BC NPs in the RPMI medium. The insets in Figure S3A show representative images of the sample at the beginning of the stirring process (Figure S3A: blue-framed inset) and after 48 h (Figure S3A: red-framed inset), where the color changes from light purple to black due to the dispersion of the BC NPs in the SABM. Additional images of BC NPs being stirred over time in SABM are provided in Figure S2. 

The cell culture medium and, specifically, the protein content can have a direct effect on the dispersion formation and its stability, as the protein’s adsorption is directly affected by its concentration in the solution [77]. The stability measurements for the BC NP-SABM system are shown in Figure S4. Similar to the dispersion in the RPMI medium, the samples of BC NPs in SABM stirred for (A) 6, (B) 12, (C) 24, (D) 48, and (E) 72 h were analyzed by DLS before and after the 24 h storage to study the stability of the dispersion reflected in the PSD variability and sample polydispersity. The Z-Average values together with the PDI values for BC NPs in SABM are summarized in Table S3. Moreover, the zeta potential and the conductivity of BC NPs’ dispersion in SABM are provided in Table S2. At 6 h, the PSD varied significantly, indicating low dispersion stability. At this point, a significant amount of the BC NPs was still captured in the foam (Figure S2) and, therefore, the BC NPs could not be properly stabilized. With increasing stirring time, the deviations in the PSD measured by DLS decreased. The smallest PSD variability was detected after 48 h (Figure S4D). Therefore, it is concluded that for this NP–culture medium system, a 48 h stirring suffices to form a proper dispersion.

### 3.4. Effective NP Density and Dosimetry

**Figure 5:**
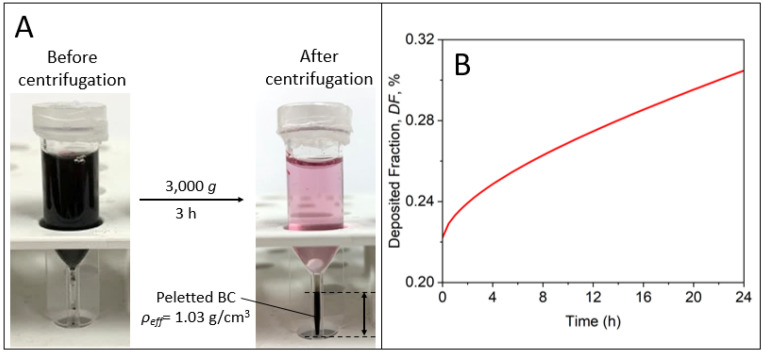
Effective density, *ρ_eff_*, of BC NPs in RPMI medium established by the VCM method (**A**) and the deposited fraction calculated by the DG model (**B**).

Effective densities, *ρ_eff_*, of 1.03 and 1.07 g/cm^3^ were measured for BC NPs dispersed in RPMI medium (Figure 5A) and SABM (Figure S5A), respectively. The *ρ_eff_* values measured here align well with the data previously measured for the engineered carbon-based NPs, where the values of 1.02 g/cm^3^ and 1.04 g/cm^3^ were measured for Carbon Nanohorns and Printex-90 (carbon black) NPs, respectively [31]. 

The value of *ρ_eff_* along with the entire PSD were used to calculate the fraction of the NPs deposited on the cells (Figure 5B and Figure S5B), which was shown to be below 0.5% in the cases of both studied media at the end of a 24 h exposure period. This very low NP cell deposition as a function of time is due to the very low effective density of the formed agglomerates [31]. High-density NPs like CeO_2_ (bulk density, *ρ_b_* = 7.22 g/cm^3^, *ρ_eff_* = 1.44 g/cm^3^), Fe_2_O_3_ (*ρ_b_* = 5.25 g/cm^3^, *ρ_eff_* = 1.91 g/cm^3^), or Cr_2_O_3_ (*ρ_b_* = 5.22 g/cm^3^, *ρ_eff_* = 2.21 g/cm^3^) deposited on the cells very quickly and, therefore, the deposited fraction was able to exceed 90% within the 24 h [31]. On the other hand, buoyant NPs (e.g., polypropylene, *ρ_b_* = *ρ_eff_* = 0.9 g/cm^3^) [78] with densities lower than that of the media tend to accumulate close to the top of the liquid column and must be studied using inverted cell culture platforms as previously described by the authors [78]. It is worth noting that alternative approaches can be used for low-density, slowly settling nanoparticles, which may include the use of rotation of the cell culture system containing particles and cells suspended in the medium [79,80]. This, however, does not apply to the case of adherent cells. 

So, NPs with effective densities close to the media density, such as hydrophilic flame-made silica (*ρ_b_* = 2.2 g/cm^3^, *ρ_eff_* = 1.12 g/cm^3^) [81] and the hydrophobic BC used here (*ρ_b_* = 1.8 g/cm^3^, *ρ_eff_* = 1.03–1.07 g/cm^3^), are expected to deposit on the cells very slowly, mostly by diffusion [82], resulting in low deposited fractions. This slow NP deposition over time may create difficulty in performing dose–response studies, and delivery of the desired dose to the cells should match physiologically relevant doses based on the route of exposure (i.e., inhalation, ingestion). In order to increase the delivered dose, the initial administered concentration of the NPs could be increased. This can, however, lead to several undesired effects in cases wherein the materials release chemical compounds from their surfaces, as their concentrations would increase and potentially cause toxic effects to the cellular system [83]. 

## 4. Conclusions

In this study, a robust methodology for preparing stable dispersions of hydrophobic nanoparticles (NPs) in cell culture media is presented based on continuous stirring in the culture medium. The versatility and applicability of the proposed dispersion preparation approach are showcased using model hydrophobic black carbon (BC) NPs and two cell culture media widely used in in vitro studies, namely (i) RPMI medium supplemented with 10% FBS, and (ii) SABM supplemented with 1% BSA. The proteins in the medium form a protective corona around the NPs, enabling their dispersion in the aqueous environment. Our findings confirm that the stirring time (dispersion energy) is NP–culture medium-specific. 

The volumetric centrifugation method revealed that BC NPs in RPMI medium and SABM have effective densities of 1.03 g/cm^3^ and 1.07 g/cm^3^, respectively, which are very close to the densities of the media and, therefore, such NPs will be delivered to the cells slowly. The slow sedimentation of NPs with effective densities close to that of the media brings challenges in designing dose–response studies. So, the data and methodology presented here will enable researchers to consider the dosimetrics in their studies.

In conclusion, the methodology presented in this study offers a valuable approach for preparing stable dispersions of hydrophobic NPs in culture media for in vitro cellular studies. The proposed approach can improve the toxicological evaluation and interlaboratory reproducibility related to hydrophobic NPs, increase the overall knowledge in the field of nanotoxicology, and enable understanding the of possible implications for public health.

## Figures and Tables

**Table 1:** Z-Averages and polydispersity indexes (PDIs) of BC NPs stirred in RPMI medium from the data shown in Figure 4.

BC in RPMI Stirred for	Immediately after Stirring		Stability Testing (24 h)	
	Z-Average (nm)	PDI	Z-Average (nm)	PDI
6 h	347 ± 10	0.573 ± 0.011	378 ± 22	0.489 ± 0.047
12 h	345 ± 25	0.484 ± 0.041	357 ± 6	0.474 ± 0.045
24 h	329 ± 12	0.430 ± 0.031	326 ± 9	0.487 ± 0.066
48 h	347 ± 5	0.425 ± 0.031	332 ± 6	0.413 ± 0.010
72 h	331 ± 2	0.426 ± 0.016	338 ± 2	0.433 ± 0.016

## Data Availability

Data are contained within the article and Supplementary Materials.

## Supplementary Materials

The following supporting information can be downloaded at: https://www.mdpi.com/article/10.3390/nano14070589/s1. Figure S1. Water Contact Angle (WCA) measured with (a) a high precision optical measuring device (OCA35, DataPhysics) and (b) the custom goniometer used in this work. Table S1. Summary of the parameters used for the particle dosimetry calculation by the DG model. The viscosities of the media were measured by a Cannon Fenske viscometer for water-based solutions, submerged in a heating bath equilibrated to 37 °C. The densities were measured by weighing a known volume of the medium pre-warmed to 37 °C in a heating bath. Figure S2. Representative images of BC NPs dispersed in RPMI (top) and SABM (bottom) after stirring for 0-72 h along with the respective Zeta-Average (Z-Average) measured by Dynamic Light Scattering (DLS). Figure S3. Representative images (A) of the dispersions of BC NPs in SABM medium after stirring for 0 (blue-framed) and 48 h (red-framed), along with intensity-based particle size distributions measured by DLS for the medium only (dot-broken line) or BC dispersions after 0 (B), 6 (C), 12 (D), 24 (E), 72 (F) or 48 h (broken line). Variation within three DLS measurements of the sample stirred for 48 h is quantified by the red shaded area. Table S2. Zeta potential and conductivity of BC NPs dispersed in RPMI and SABM media measured by DLS for stable dispersions obtained at 24 h and 48 h of stirring, respectively. Figure S4. Stability of the BC size distribution measured in SABM medium right after stirring (broken lines) for 6 (A), 12 (B), 24 (C), 48 (D) and 72 h (E) or after 24 h of storage at room temperature (solid lines). The errorbars quantify the statistical variation over 3 measurements. Table S3. Z-Average and polydispersity index (PDI) of BC NPs stirred in SABM medium from the data shown in Figure S4. Figure S5. Effective density, ρeff, of BC NPs in SABM medium established by VCM method (A) and the deposited fraction calculated by the DG model (B).

## References

[1] Nel A., Xia T., Madler L., Li N. (2006). Toxic potential of materials at the nanolevel. Science.

[2] Van den Broek J., Abegg S., Pratsinis S.E., Güntner A.T. (2019). Highly selective detection of methanol over ethanol by a handheld gas sensor. Nat. Commun..

[3] Abegg S., Magro L., van den Broek J., Pratsinis S.E., Güntner A.T. (2020). A pocket-sized device enables detection of methanol adulteration in alcoholic beverages. Nat. Food.

[4] Farjadian F., Ghasemi A., Gohari O., Roointan A., Karimi M., Hamblin M.R. (2019). Nanopharmaceuticals and nanomedicines currently on the market: Challenges and opportunities. Nanomedicine.

[5] Wilhelm S., Tavares A.J., Dai Q., Ohta S., Audet J., Dvorak H.F., Chan W.C.W. (2016). Analysis of nanoparticle delivery to tumours. Nat. Rev. Mater..

[6] Servin A.D., White J.C. (2016). Nanotechnology in agriculture: Next steps for understanding engineered nanoparticle exposure and risk. NanoImpact.

[7] Das M., Saxena N., Dwivedi P.D. (2009). Emerging trends of nanoparticles application in food technology: Safety paradigms. Nanotoxicology.

[8] Lewinski N., Colvin V., Drezek R. (2008). Cytotoxicity of nanoparticles. Small.

[9] Madl A.K., Pinkerton K.E. (2009). Health effects of inhaled engineered and incidental nanoparticles. Crit. Rev. Toxicol..

[10] Yeganeh B., Kull C.M., Hull M.S., Marr L.C. (2008). Characterization of airborne particles during production of carbonaceous nanomaterials. Environ. Sci. Technol..

[11] Sotiriou G.A., Pratsinis S.E. (2011). Engineering nanosilver as an antibacterial, biosensor and bioimaging material. Curr. Opin. Chem. Eng..

[12] Benn T.M., Westerhoff P. (2008). Nanoparticle silver released into water from commercially available sock fabrics. Environ. Sci. Technol..

[13] Wohlleben W., Meyer J., Muller J., Mueller P., Vilsmeier K., Stahlmecke B., Kuhlbusch T.A. (2016). Release from nanomaterials during their use phase: Combined mechanical and chemical stresses applied to simple and multi-filler nanocomposites mimicking wear of nano-reinforced tires. Environ. Sci. Nano.

[14] Kittelson D.B. (1998). Engines and nanoparticles: A review. J. Aerosol Sci..

[15] Park K., Kittelson D.B., McMurry P.H. (2004). Structural properties of diesel exhaust particles measured by transmission electron microscopy (TEM): Relationships to particle mass and mobility. Aerosol Sci. Technol..

[16] Corbin J.C., Mensah A.A., Pieber S.M., Orasche J., Michalke B., Zanatta M., Czech H., Massabo D., de Mongeot F.B., Mennucci C. (2018). Trace Metals in Soot and PM_2.5_ from Heavy-Fuel-Oil Combustion in a Marine Engine. Environ. Sci. Technol..

[17] Lack D.A., Corbett J.J. (2012). Black carbon from ships: A review of the effects of ship speed, fuel quality and exhaust gas scrubbing. Atmos. Chem. Phys..

[18] Petzold A., Dopelheuer A., Brock C.A., Schroder F. (1999). In situ observations and model calculations of black carbon emission by aircraft at cruise altitude. J. Geophys. Res.-Atmos..

[19] Stettler M.E.J., Boies A.M., Petzold A., Barrett S.R.H. (2013). Global Civil Aviation Black Carbon Emissions. Environ. Sci. Technol..

[20] Xu R., Ye T., Yue X., Yang Z., Yu W., Zhang Y., Bell M.L., Morawska L., Yu P., Zhang Y. (2023). Global population exposure to landscape fire air pollution from 2000 to 2019. Nature.

[21] Burke M., Childs M.L., de la Cuesta B., Qiu M., Li J., Gould C.F., Heft-Neal S., Wara M. (2023). The contribution of wildfire to PM_2. 5_ trends in the USA. Nature.

[22] Nim N., Morris J., Tekasakul P., Dejchanchaiwong R. (2023). Fine and ultrafine particle emission factors and new diagnostic ratios of PAHs for peat swamp forest fires. Environ. Pollut..

[23] Jia J., Wang Z., Yue T., Su G., Teng C., Yan B. (2020). Crossing biological barriers by engineered nanoparticles. Chem. Res. Toxicol..

[24] Pietroiusti A., Campagnolo L., Fadeel B. (2013). Interactions of engineered nanoparticles with organs protected by internal biological barriers. Small.

[25] Fournier S.B., D’Errico J.N., Adler D.S., Kollontzi S., Goedken M.J., Fabris L., Yurkow E.J., Stapleton P.A. (2020). Nanopolystyrene translocation and fetal deposition after acute lung exposure during late-stage pregnancy. Part. Fibre Toxicol..

[26] Pang Y., Qu J., Zhang H., Cao Y., Ma X., Wang S., Wang J., Wu J., Zhang T. (2023). Nose-to-brain translocation and nervous system injury in response to indium tin oxide nanoparticles of long-term low-dose exposures. Sci. Total Environ..

[27] Joris F., Manshian B.B., Peynshaert K., De Smedt S.C., Braeckmans K., Soenen S.J. (2013). Assessing nanoparticle toxicity in cell-based assays: Influence of cell culture parameters and optimized models for bridging the in vitro–in vivo gap. Chem. Soc. Rev..

[28] Garza K.M., Soto K.F., Murr L.E. (2008). Cytotoxicity and reactive oxygen species generation from aggregated carbon and carbonaceous nanoparticulate materials. Int. J. Nanomed..

[29] Brunner T.J., Wick P., Manser P., Spohn P., Grass R.N., Limbach L.K., Bruinink A., Stark W.J. (2006). In vitro cytotoxicity of oxide nanoparticles: Comparison to asbestos, silica, and the effect of particle solubility. Environ. Sci. Technol..

[30] Elsaesser A., Howard C.V. (2012). Toxicology of nanoparticles. Adv. Drug Deliv. Rev..

[31] Cohen J.M., Teeguarden J.G., Demokritou P. (2014). An integrated approach for the in vitro dosimetry of engineered nanomaterials. Part. Fibre Toxicol..

[32] DeLoid G.M., Cohen J.M., Pyrgiotakis G., Pirela S.V., Pal A., Liu J., Srebric J., Demokritou P. (2015). Advanced computational modeling for in vitro nanomaterial dosimetry. Part. Fibre Toxicol..

[33] Hinderliter P.M., Minard K.R., Orr G., Chrisler W.B., Thrall B.D., Pounds J.G., Teeguarden J.G. (2010). ISDD: A computational model of particle sedimentation, diffusion and target cell dosimetry for in vitro toxicity studies. Part. Fibre Toxicol..

[34] DeLoid G.M., Cohen J.M., Pyrgiotakis G., Demokritou P. (2017). Preparation, characterization, and in vitro dosimetry of dispersed, engineered nanomaterials. Nat. Protoc..

[35] Wang J.L., Xu L.J. (2012). Advanced oxidation processes for wastewater treatment: Formation of hydroxyl radical and application. Crit. Rev. Environ. Sci. Technol..

[36] Miljevic B., Hedayat F., Stevanovic S., Fairfull-Smith K., Bottle S., Ristovski Z. (2014). To sonicate or not to sonicate PM filters: Reactive oxygen species generation upon ultrasonic irradiation. Aerosol Sci. Technol..

[37] Riesz P., Kondo T. (1992). Free radical formation induced by ultrasound and its biological implications. Free Radic. Biol. Med..

[38] Yan Y.-L., Cai Y.-X., Liu X.-C., Ma G.-W., Lv W., Wang M.-X. (2020). Hydrophobic modification on the surface of SiO_2_ nanoparticle: Wettability control. Langmuir.

[39] Wang H., Fu Z., Ji X., Lu M., Deng L., Liu Z., Yin B., Ni D. (2023). A general method for endowing hydrophobic nanoparticles with water dispersion abilities. J. Mater. Chem. B.

[40] Dederichs T., Moller M., Weichold O. (2009). Colloidal stability of hydrophobic nanoparticles in ionic surfactant solutions: Definition of the critical dispersion concentration. Langmuir.

[41] Don Porto Carero A., Hoet P., Verschaeve L., Schoeters G., Nemery B. (2001). Genotoxic effects of carbon black particles, diesel exhaust particles, and urban air particulates and their extracts on a human alveolar epithelial cell line (A549) and a human monocytic cell line (THP-1). Environ. Mol. Mutagen..

[42] Coreas R., Castillo C., Li Z., Yan D., Gao Z., Chen J., Bitounis D., Parviz D., Strano M.S., Demokritou P. (2022). Biological Impacts of Reduced Graphene Oxide Affected by Protein Corona Formation. Chem. Res. Toxicol..

[43] Stone V., Shaw J., Brown D., MacNee W., Faux S., Donaldson K. (1998). The role of oxidative stress in the prolonged inhibitory effect of ultrafine carbon black on epithelial cell function. Toxicol. Vitr..

[44] Stathopulos P.B., Scholz G.A., Hwang Y.M., Rumfeldt J.A., Lepock J.R., Meiering E.M. (2004). Sonication of proteins causes formation of aggregates that resemble amyloid. Protein Sci..

[45] Chen R.J., Bangsaruntip S., Drouvalakis K.A., Wong Shi Kam N., Shim M., Li Y., Kim W., Utz P.J., Dai H. (2003). Noncovalent functionalization of carbon nanotubes for highly specific electronic biosensors. Proc. Natl. Acad. Sci. USA.

[46] Deguchi S., Yamazaki T., Mukai S.-A., Usami R., Horikoshi K. (2007). Stabilization of C60 nanoparticles by protein adsorption and its implications for toxicity studies. Chem. Res. Toxicol..

[47] Raffaini G., Ganazzoli F. (2003). Simulation study of the interaction of some albumin subdomains with a flat graphite surface. Langmuir.

[48] Tenzer S., Docter D., Kuharev J., Musyanovych A., Fetz V., Hecht R., Schlenk F., Fischer D., Kiouptsi K., Reinhardt C. (2013). Rapid formation of plasma protein corona critically affects nanoparticle pathophysiology. Nat. Nanotechnol..

[49] Maiorano G., Sabella S., Sorce B., Brunetti V., Malvindi M.A., Cingolani R., Pompa P.P. (2010). Effects of cell culture media on the dynamic formation of protein-nanoparticle complexes and influence on the cellular response. ACS Nano.

[50] Kopac T. (2021). Protein corona, understanding the nanoparticle–protein interactions and future perspectives: A critical review. Int. J. Biol. Macromol..

[51] Bilardo R., Traldi F., Vdovchenko A., Resmini M. (2022). Influence of surface chemistry and morphology of nanoparticles on protein corona formation. Wiley Interdiscip. Rev. Nanomed. Nanobiotechnol..

[52] Owens III D.E., Peppas N.A. (2006). Opsonization, biodistribution, and pharmacokinetics of polymeric nanoparticles. Int. J. Pharm..

[53] Schvartz M., Saudrais F., Devineau S., Chédin S., Jamme F., Leroy J., Rakotozandriny K., Taché O., Brotons G., Pin S. (2023). Role of the Protein Corona in the Colloidal Behavior of Microplastics. Langmuir.

[54] Trivanovic U., Kelesidis G.A., Pratsinis S.E. (2022). High-throughput generation of aircraft-like soot. Aerosol Sci. Technol..

[55] Trivanovic U., Pereira Martins M., Benz S., Kelesidis G.A., Pratsinis S.E. (2023). Dynamics of soot surface growth and agglomeration by enclosed spray combustion of jet fuel. Fuel.

[56] Lamour G., Hamraoui A., Buvailo A., Xing Y., Keuleyan S., Prakash V., Eftekhari-Bafrooei A., Borguet E. (2010). Contact angle measurements using a simplified experimental setup. J. Chem. Educ..

[57] Schneider C.A., Rasband W.S., Eliceiri K.W. (2012). NIH Image to ImageJ: 25 years of image analysis. Nat. Methods.

[58] Yon J., Bescond A., Liu F. (2015). On the radiative properties of soot aggregates part 1: Necking and overlapping. J. Quant. Spectrosc. Radiat. Transf..

[59] DeLoid G., Cohen J.M., Darrah T., Derk R., Rojanasakul L., Pyrgiotakis G., Wohlleben W., Demokritou P. (2014). Estimating the effective density of engineered nanomaterials for in vitro dosimetry. Nat. Commun..

[60] Song C., Wang P., Makse H.A. (2008). A phase diagram for jammed matter. Nature.

[61] Cheimarios N., Pem B., Tsoumanis A., Ilić K., Vrček I.V., Melagraki G., Bitounis D., Isigonis P., Dusinska M., Lynch I. (2022). An In Vitro Dosimetry Tool for the Numerical Transport Modeling of Engineered Nanomaterials Powered by the Enalos RiskGONE Cloud Platform. Nanomaterials.

[62] Thomas D.G., Smith J.N., Thrall B.D., Baer D.R., Jolley H., Munusamy P., Kodali V., Demokritou P., Cohen J., Teeguarden J.G. (2018). ISD3: A particokinetic model for predicting the combined effects of particle sedimentation, diffusion and dissolution on cellular dosimetry for in vitro systems. Part. Fibre Toxicol..

[63] Kelesidis G.A., Rossi N., Pratsinis S.E. (2022). Porosity and crystallinity dynamics of carbon black during internal and surface oxidation. Carbon.

[64] Parent P., Laffon C., Marhaba I., Ferry D., Regier T., Ortega I., Chazallon B., Carpentier Y., Focsa C. (2016). Nanoscale characterization of aircraft soot: A high-resolution transmission electron microscopy, Raman spectroscopy, X-ray photoelectron and near-edge X-ray absorption spectroscopy study. Carbon.

[65] Tsai P.-J., Shieh H.-Y., Lee W.-J., Lai S.-O. (2001). Health-risk assessment for workers exposed to polycyclic aromatic hydrocarbons (PAHs) in a carbon black manufacturing industry. Sci. Total Environ..

[66] Lighty J.S., Veranth J.M., Sarofim A.F. (2000). Combustion aerosols: Factors governing their size and composition and implications to human health. J. Air Waste Manag. Assoc..

[67] Scheepers P., Bos R. (1992). Combustion of diesel fuel from a toxicological perspective: I. Origin of incomplete combustion products. Int. Arch. Occup. Environ. Health.

[68] Jeannet N., Fierz M., Kalberer M., Burtscher H., Geiser M. (2015). Nano aerosol chamber for in-vitro toxicity (NACIVT) studies. Nanotoxicology.

[69] Jonsdottir H.R., Delaval M., Leni Z., Keller A., Brem B.T., Siegerist F., Schönenberger D., Durdina L., Elser M., Burtscher H. (2019). Non-volatile particle emissions from aircraft turbine engines at ground-idle induce oxidative stress in bronchial cells. Commun. Biol..

[70] Delaval M.N., Jonsdottir H.R., Leni Z., Keller A., Brem B.T., Siegerist F., Schönenberger D., Durdina L., Elser M., Salathe M. (2022). Responses of reconstituted human bronchial epithelia from normal and health-compromised donors to non-volatile particulate matter emissions from an aircraft turbofan engine. Environ. Pollut..

[71] Pierdominici M., Maselli A., Cecchetti S., Tinari A., Mastrofrancesco A., Alfè M., Gargiulo V., Beatrice C., Di Blasio G., Carpinelli G. (2014). Diesel exhaust particle exposure in vitro impacts T lymphocyte phenotype and function. Part. Fibre Toxicol..

[72] Kanungo M., Mettu S., Law K.-Y., Daniel S. (2014). Effect of Roughness Geometry on Wetting and Dewetting of Rough PDMS Surfaces. Langmuir.

[73] Wei Y., Zhang Q., Thompson J.E. (2017). The wetting behavior of fresh and aged soot studied through contact angle measurements. Atmos. Clim. Sci..

[74] Xu L., Karunakaran R.G., Guo J., Yang S. (2012). Transparent, superhydrophobic surfaces from one-step spin coating of hydrophobic nanoparticles. ACS Appl. Mater. Interfaces.

[75] Bayer I.S., Steele A., Loth E. (2013). Superhydrophobic and electroconductive carbon nanotube-fluorinated acrylic copolymer nanocomposites from emulsions. Chem. Eng. J..

[76] Li X., He F., Hu S., Sun N., Huo C., Liu R. (2023). The culprits of superoxide dismutase inactivation under size-dependent stress of ultrafine carbon black: Superoxide anion, genotoxicity and protein corona. Sci. Total Environ..

[77] Dee K.C., Puleo D.A., Bizios R. (2003). An Introduction to Tissue-Biomaterial Interactions.

[78] Watson C., DeLoid G., Pal A., Demokritou P. (2016). Buoyant nanoparticles: Implications for nano-biointeractions in cellular studies. Small.

[79] Rader C.P., Sterner T., Jakob F., Schütze N., Eulert J. (1999). Cytokine response of human macrophage-like cells after contact with polyethylene and pure titanium particles. J. Arthroplast..

[80] Matsusaki T., Kawanabe K., Ise K., Nakayama T., Toguchida J., Nakamura T. (2007). Gene expression profile of macrophage-like U937 cells in response to polyethylene particles: A novel cell-particle culture system. J. Arthroplast..

[81] Rubio L., Pyrgiotakis G., Beltran-Huarac J., Zhang Y., Gaurav J., Deloid G., Spyrogianni A., Sarosiek K.A., Bello D., Demokritou P. (2019). Safer-by-design flame-sprayed silicon dioxide nanoparticles: The role of silanol content on ROS generation, surface activity and cytotoxicity. Part. Fibre Toxicol..

[82] Teeguarden J.G., Hinderliter P.M., Orr G., Thrall B.D., Pounds J.G. (2007). Particokinetics in vitro: Dosimetry considerations for in vitro nanoparticle toxicity assessments. Toxicol. Sci..

[83] Misra S.K., Dybowska A., Berhanu D., Luoma S.N., Valsami-Jones E. (2012). The complexity of nanoparticle dissolution and its importance in nanotoxicological studies. Sci. Total Environ..

