# Supplementary material for: Dispersion and Dosimetric Challenges of Hydrophobic Carbon-Based Nanoparticles in In Vitro Cellular Studies
Source: Nanomaterials (Basel). 2024 Mar 27;14(7):589. doi: 10.3390/nano14070589 (PMC11013865; doi:10.3390/nano14070589)
Supplement: Supplementary file 1 [file nanomaterials-14-00589-s001.zip › nanomaterials-2877094-supplementary.pdf]

Supplementary information for

Dispersion and Dosimetric Challenges of Hydrophobic Carbon-Based Nanoparticles in In Vitro Cellular Studies

Denisa Lizonova[1], *Una Trivanovic*[2], *Philip Demokritou*[1,*], *Georgios. A. Kelesidis*[1,2*]

[1] Nanoscience and Advanced Materials Center (NAMC), Environmental and Occupational Health Science Institute, School of Public Health, Rutgers University, 170 Frelinghuysen Road, Piscataway, NJ, 08854, USA

[2] Particle Technology Laboratory, Institute of Energy and Process Engineering, Department of Mechanical and Process Engineering, ETH Zürich, Sonneggstrasse 3, CH-8092, Zürich, Switzerland

*Corresponding authors: philip.demokritou@rutgers.edu, georgios.kelesidis@rutgers.edu

8 Pages

3 Tables

5 Figures

Summary

Figure S1 .... p. S3

S2 .... p. S5

S3 .... p. S6

S4 .... p. S7

S5 .... p. S8

Table S1 .... p. S4

S2 .... p. S6

S3 .... p. S7

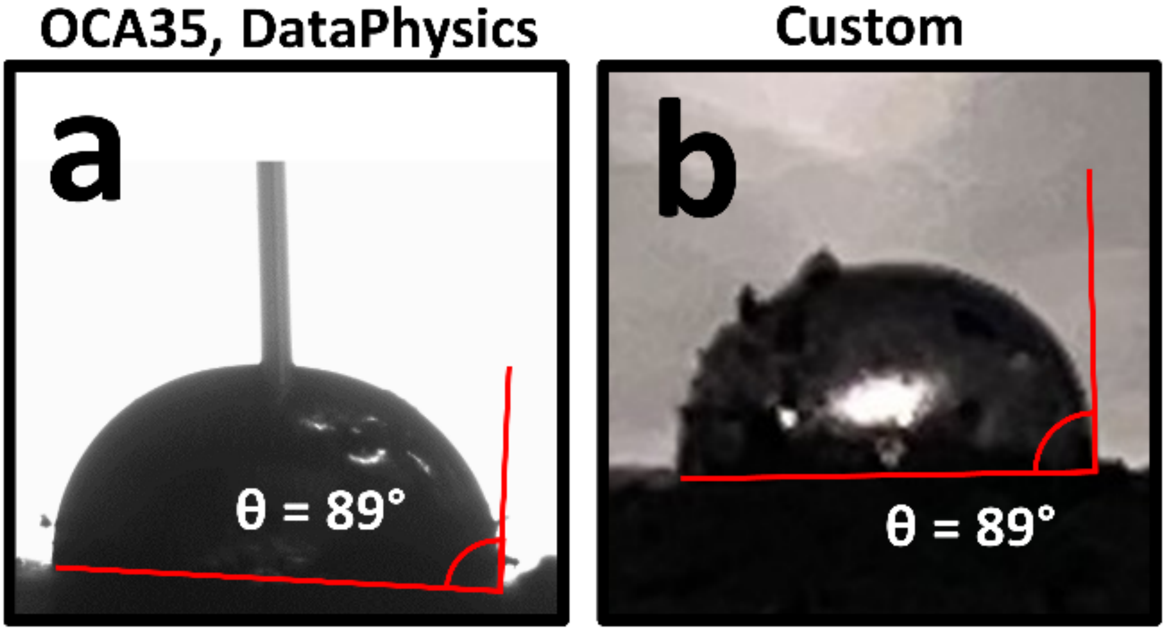

Figure S1. Water Contact Angle (WCA) measured with a) a high precision optical measuring device (OCA35, DataPhysics) and b) the custom goniometer used in this work.

Table S1. Summary of the parameters used for the particle dosimetry calculation by the DG model. The viscosities of the media were measured by a Cannon Fenske viscometer for water-based solutions, submerged in a heating bath equilibrated to 37 °C. The densities were measured by weighing a known volume of the medium pre-warmed to 37 °C in a heating bath.

		RPMI	SABM
Particle Parameters	Material	User defined	
	Density	1.8 g/cm^3	
	Distribution type	Fraction distribution by volume	
	Effective density	1.03 g/cm^3	1.07 g/cm^3
Solvent Parameters	Density	1.0104 g/cm^3	0.995 g/cm^3
	Viscosity	0.0081 P	0.0075 P
	Temperature	37 °C	37 °C
Simulation Parameters	Suspension column height	4.5 mm	
	Height of subcompartment	0.005 mm	
	Initial total concentration of material	0.1 mg/cm^3	
	Centrifugation	1 (gravity)	
	Total time of simulation	24 h	
	Time interval for simulation	0.5 s	
Output Parameters	Time interval	30 min	
	Compartment height	0.01 mm	
	Write output for...	Bottom area only	

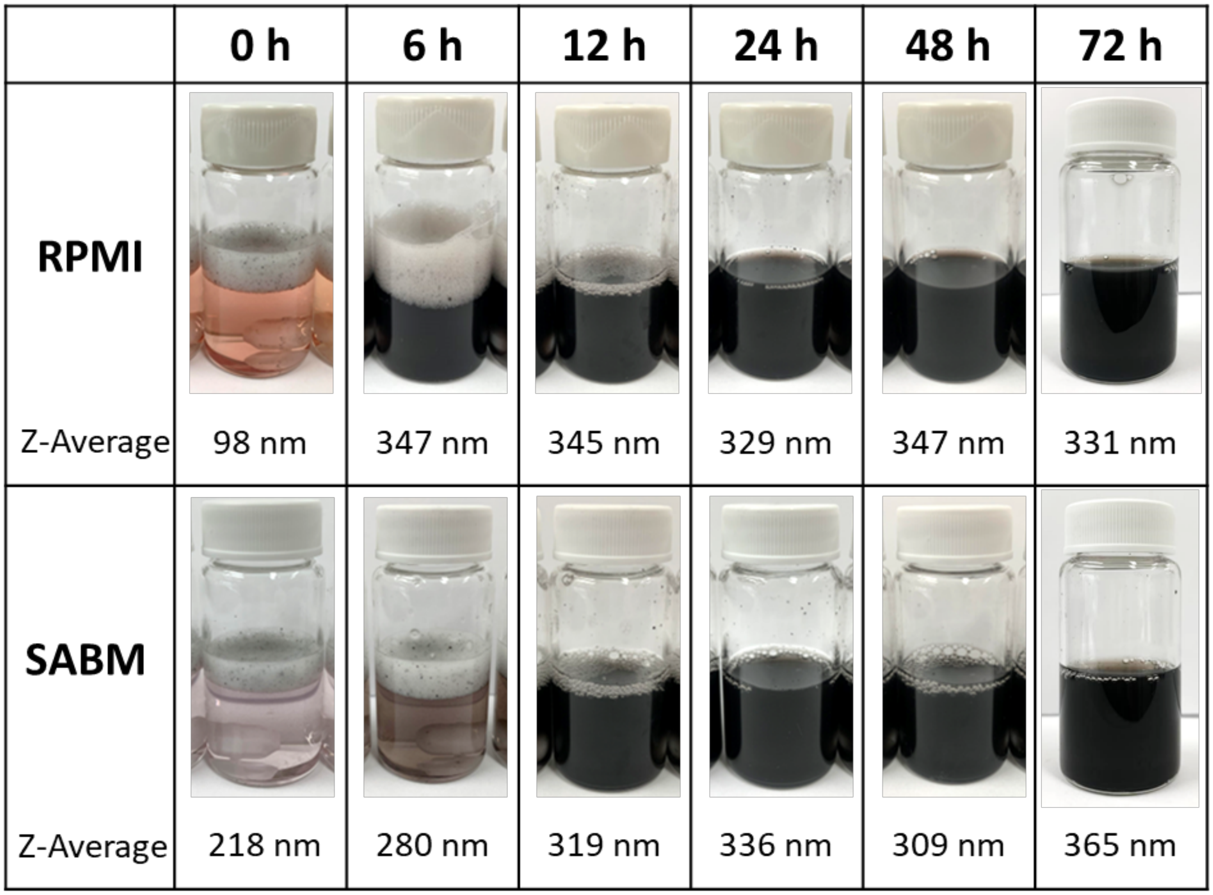

Figure S2. Representative images of BC NPs dispersed in RPMI (top) and SABM (bottom) after stirring for 0-72 h along with the respective Zeta-Average (Z-Average) measured by Dynamic Light Scattering (DLS).

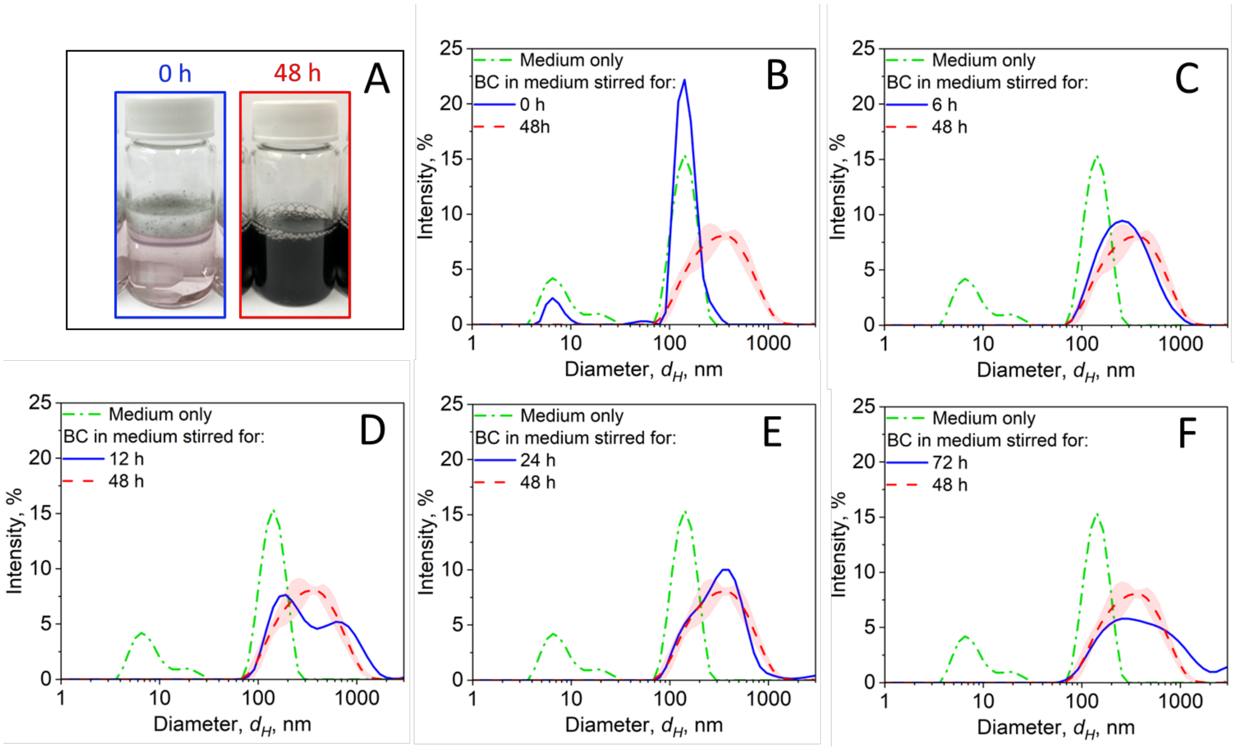

Figure S3. Representative images (A) of the dispersions of BC NPs in SABM medium after stirring for 0 (blue-framed) and 48 h (red-framed), along with intensity-based particle size distributions measured by DLS for the medium only (dot-broken line) or BC dispersions after 0 (B), 6 (C), 12 (D), 24 (E), 72 (F) or 48 h (broken line). Variation within three DLS measurements of the sample stirred for 48 h is quantified by the red shaded area.

Table S2. Zeta potential and conductivity of BC NPs dispersed in RPMI and SABM media measured by DLS for stable dispersions obtained at 24 h and 48 h of stirring, respectively.

	Zeta Potential (mV)	Conductivity (mS/cm)
BC in RPMI	-13 ± 2	15.7 ± 1.3
BC in SABM	-12 ± 1	14.2 ± 0.8

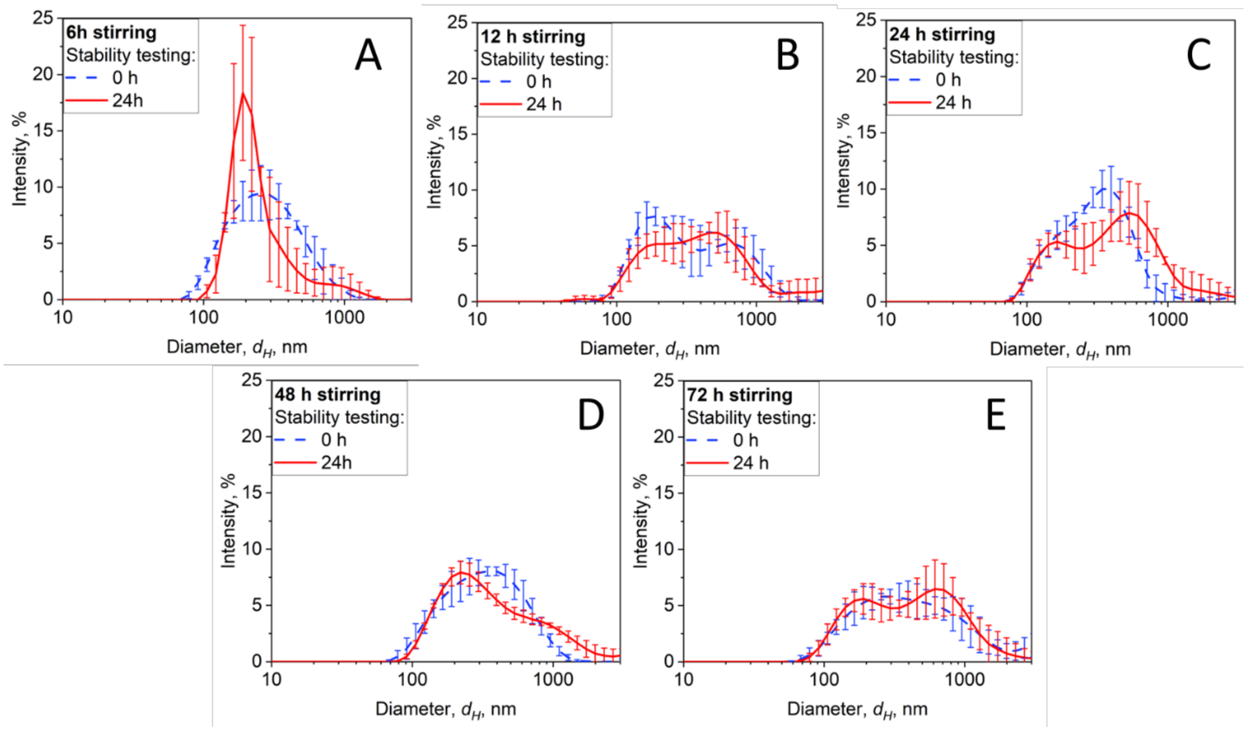

Figure S4. Stability of the BC size distribution measured in SABM medium right after stirring (broken lines) for 6 (A), 12 (B), 24 (C), 48 (D) and 72 h (E) or after 24 h of storage at room temperature (solid lines). The errorbars quantify the statistical variation over 3 measurements.

Table S3. Z-Average and polydispersity index (PDI) of BC NPs stirred in SABM medium from the data shown in Figure S4.

BC in SABM stirred for:	Immediately after stirring		Stability testing (24 h)	
	Z-Average (nm)	PDI	Z-Average (nm)	PDI
6 h	280 ± 19	0.407 ± 0.016	321 ± 54	0.469 ± 0.051
12 h	319 ± 14	0.410 ± 0.019	415 ± 6	0.735 ± 0.037
24 h	336 ± 26	0.431 ± 0.022	326 ± 33	0.456 ± 0.064
48 h	309 ± 9	0.409 ± 0.042	334 ± 12	0.492 ± 0.044
72 h	365 ± 7	0.518 ± 0.087	379 ±17	0.476 ± 0.052

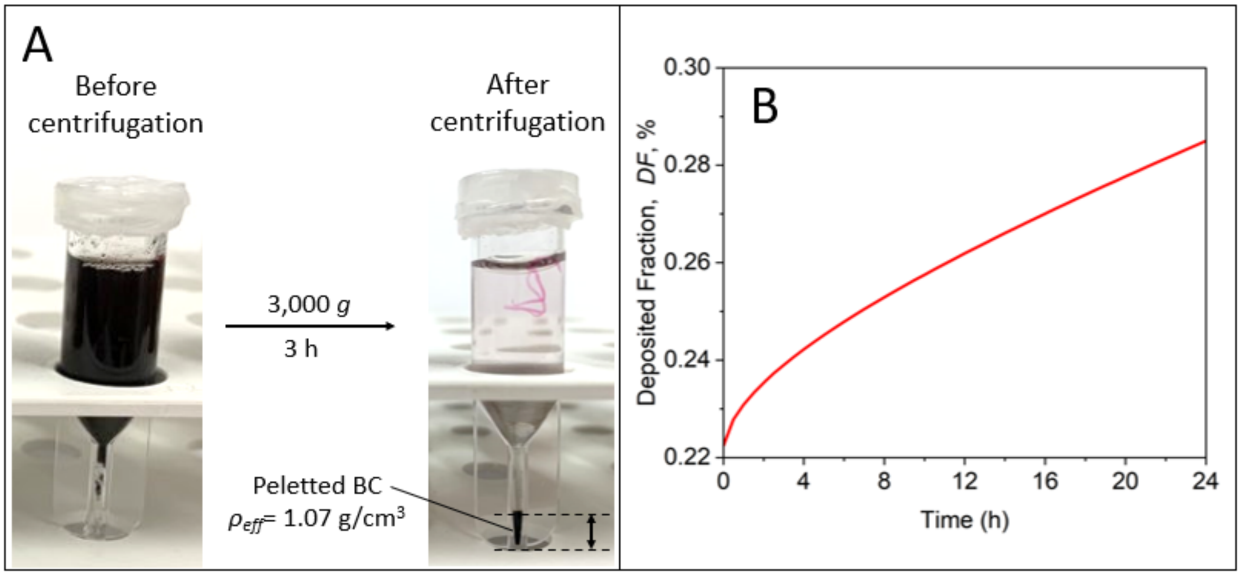

Figure S5. Effective density, ρ_{eff}, of BC NPs in SABM medium established by VCM method (A) and the deposited fraction calculated by the DG model (B).
